# Smartphone-Detected Ambient Speech and Self-Reported Measures of Anxiety and Depression: Exploratory Observational Study

**DOI:** 10.2196/22723

**Published:** 2021-01-29

**Authors:** Daniel Di Matteo, Wendy Wang, Kathryn Fotinos, Sachinthya Lokuge, Julia Yu, Tia Sternat, Martin A Katzman, Jonathan Rose

**Affiliations:** 1 The Centre for Automation of Medicine The Edward S Rogers Sr Department of Electrical and Computer Engineering University of Toronto Toronto, ON Canada; 2 START Clinic for Mood and Anxiety Disorders Toronto, ON Canada; 3 Department of Psychology Adler Graduate Professional School Toronto, ON Canada; 4 Department of Psychology Lakehead University Thunder Bay, ON Canada; 5 The Northern Ontario School of Medicine Thunder Bay, ON Canada

**Keywords:** mobile sensing, passive sensing, psychiatric assessment, mood and anxiety disorders, mobile apps, linguistics, speech recognition, speech content, lexical choice

## Abstract

**Background:**

The ability to objectively measure the severity of depression and anxiety disorders in a passive manner could have a profound impact on the way in which these disorders are diagnosed, assessed, and treated. Existing studies have demonstrated links between both depression and anxiety and the linguistic properties of words that people use to communicate. Smartphones offer the ability to passively and continuously detect spoken words to monitor and analyze the linguistic properties of speech produced by the speaker and other sources of ambient speech in their environment. The linguistic properties of automatically detected and recognized speech may be used to build objective severity measures of depression and anxiety.

**Objective:**

The aim of this study was to determine if the linguistic properties of words passively detected from environmental audio recorded using a participant’s smartphone can be used to find correlates of symptom severity of social anxiety disorder, generalized anxiety disorder, depression, and general impairment.

**Methods:**

An Android app was designed to collect periodic audiorecordings of participants’ environments and to detect English words using automatic speech recognition. Participants were recruited into a 2-week observational study. The app was installed on the participants’ personal smartphones to record and analyze audio. The participants also completed self-report severity measures of social anxiety disorder, generalized anxiety disorder, depression, and functional impairment. Words detected from audiorecordings were categorized, and correlations were measured between words counts in each category and the 4 self-report measures to determine if any categories could serve as correlates of social anxiety disorder, generalized anxiety disorder, depression, or general impairment.

**Results:**

The participants were 112 adults who resided in Canada from a nonclinical population; 86 participants yielded sufficient data for analysis. Correlations between word counts in 67 word categories and each of the 4 self-report measures revealed a strong relationship between the usage rates of death-related words and depressive symptoms (*r*=0.41, *P*<.001). There were also interesting correlations between rates of word usage in the categories of reward-related words with depression (*r*=–0.22, *P*=.04) and generalized anxiety (*r*=–0.29, *P*=.007), and vision-related words with social anxiety (*r*=0.31, *P*=.003).

**Conclusions:**

In this study, words automatically recognized from environmental audio were shown to contain a number of potential associations with severity of depression and anxiety. This work suggests that sparsely sampled audio could provide relevant insight into individuals’ mental health.

## Introduction

### Background

Depression and anxiety disorders are mental health conditions that can, and do, impact people from all geographic and socioeconomic areas of life. Those who suffer from these disorders experience a lower quality of life [1], and many people unknowingly suffer from these disorders due to lack of sufficient access to mental health care or misdiagnoses [2]. The challenge presented by these disorders requires efforts in many areas, including improvements to policy, funding, outreach, treatment, and pharmacotherapy, among others. The diagnosis and assessment of depression and anxiety disorders is also an area where improvements may reduce suffering and improve quality of life for those living with the disorders. In this paper, we explore how fine-grained technology-enhanced observation of patients might give insights into their mental health state.

Modern smartphones are ubiquitous devices that are equipped with a number of sensors that can sense physical activity, geolocation, communication patterns, and the speech of their owners as they go about their day-to-day lives. This sensing capability offers a potential new paradigm for diagnosis and assessment, where instead of asking patients to report their feelings and behaviors relevant to their mental health, it might be possible to infer this information passively and objectively from smartphone-collected data [3]. Given enough data over time, these inferences may prove sufficient to act as a novel severity measure for depression and anxiety disorders. A key advantage of this approach would be that these severity measures would not require expensive, unavailable, or otherwise inaccessible mental health professionals. This study focused specifically on how the linguistic content of speech, recognized from ambient audio recorded by participants’ smartphones, may be used as correlates of severity of depression, anxiety, and impairment due to poor mental health.

### Prior Work

Our prior efforts explored audio (nonlinguistic) features and correlates with mental health scales [4].

The link between the words spoken by an individual and anxiety or depression has been investigated in 2 major subdomains. The first is the acoustic features of words, that is, the qualities and characteristics of the sounds produced independent of the meaning of the words spoken. While not the focus of this work, prior work has demonstrated numerous quantifiable differences in the acoustic properties of speech in depressed individuals [5]. The literature also shows links between voice acoustics and anxiety [6,7].

The second subdomain upon which this work focused, linguistic analysis, encompasses how an individual’s choice of words may relate to symptoms of depression and anxiety. Given this focus, the analysis of the written word and its relationship to anxiety and depression is just as relevant as the spoken word, as the methods employed in this study ignore the additional acoustic information present in the spoken word.

The analysis of speech content and word selection, sometimes referred to as content analysis in the literature, has been studied extensively in psychotherapy contexts [8]. Oxman et al [9] demonstrated that the analysis of speech transcripts of free-form speech could be used to classify psychiatric patients into their respective diagnostic groups with accuracy on par with psychiatric raters. Similar analysis of linguistic style has also been shown to discern between psychiatric inpatients and healthy controls—psychiatric patients used fewer words pertaining to optimism compared to controls (among other differences) [10].

In the linguistic analysis of depression, it has been widely reported that first-person singular pronoun use is correlated with depression severity. A meta-analysis of 21 studies of these correlations confirmed this relationship, where the studies performed analyses of multiple media, including writing, speech, and Facebook status updates [11]. It is believed that this relationship is as a result of the link between depression and self-focused attention [12]. A link between first-person singular pronoun use and social anxiety disorder was also demonstrated [13]. Another linguistic analysis of social anxiety disorder showed that individuals with social anxiety disorder used more positive emotion words than individuals in the control group [14]; the authors hypothesized that such behavior may be a result of the desire to appease others in the effort avoid scrutiny, which is a key fear of social anxious individuals. A number of studies [15] have mined data from social media networks (eg, Twitter) to extract linguistic features which have then been showed to capable to distinguish individuals with mental disorder (eg, depression) from neurotypical controls.

### Goal of This Study

While studies [11-15] have demonstrated links between the choice of participants’ words and mental health state, the linguistic content of their entire audio environment may shed even more light into mental states, since the environment also contains words spoken by others, such as members of conversations or speakers in news or entertainment media present in the auditory environment. The goal of this exploratory study was to determine if spoken words in recordings of participants’ environments may be used to find correlates of depression, social anxiety disorder, generalized anxiety disorder, and general psychiatric impairment.

## Methods

### Overview

This study used data collected in a previous study [4]. Participants were recruited from a web-based recruitment platform (Prolific [16]). Participants were not screened for the presence of any psychiatric diagnoses. The study inclusion criteria were the following: participants must (1) reside in Canada, (2) be fluent in English, (3) own an Android phone, (4) have completed at least 95% of their previous Prolific studies successfully, and (5) have previously participated in at least 20 Prolific studies. The final criterion was used to ensure that participants were proficient in using the Prolific system and were generally technology-literate. There were no exclusion criteria for the study. Participants were paid £11 (approximately US $13.37) for participating in the study.

Participants entered a 2-week observational study in which a custom app was installed onto their personal Android phone. Self-report measures of anxiety, depression, and general quality of life were collected at the beginning and end of the study. Throughout the duration of the study, the smartphone app passively collected audiorecordings of the environment (15-second recordings approximately every 5 minutes). The study was approved by the University of Toronto Health Sciences Research Ethics Board (protocol 36687).

### Materials and Data

Participants completed 4 self-report measures, in digital form within the study app, at the beginning and end of the 14-day study. A review [17] found that self-administered survey scores do not differ when deployed by app versus other delivery modes. These surveys were completed by participants on their own, with no supervision by clinicians. Participants completed the following 4 self-report measures of mental health: the Liebowitz Social Anxiety Scale (LSAS), which is a 24-item self-report scale used in the assessment of social anxiety disorder [18]; the Generalized Anxiety Disorder 7-item scale (GAD-7), which is an assessment tool for generalized anxiety disorder [19]; the Patient Health Questionnaire 8-item scale (PHQ-8), which is an assessment tool for depression [20]; and the Sheehan Disability Scale, which is a 3-item scale that assesses general impairment due to mental health [21].

The self-report scores collected at the end of the study were used for analysis because the self-report measures ask respondents to evaluate symptoms over the past 2 weeks; therefore, the window of symptom assessment would coincide with the window of electronic data collection.

To assess the severity of the exit scores, we also used the LSAS, GAD-7, and PHQ-8 scores to screen participants for social anxiety, generalized anxiety, and depression, respectively, using diagnostic thresholds found in the literature. A cutpoint of 60 [22] was used with the LSAS scores to screen for social anxiety disorder (generalized subtype). A cutpoint of 10 [19] was used with the GAD-7 scores to screen for generalized anxiety disorder. A cutpoint of 10 [20] was used with the PHQ-8 scores to screen for depression.

Spoken words detected in the participants’ environments were collected by the smartphone app. To do so, audiorecordings were collected every 5 minutes for a duration of 15 seconds by the app. These audiorecordings were captured consistently throughout the study at all hours of the day. Transcripts of the audiorecordings were generated using automatic speech recognition software (Google Speech-to-Text [23]). Transcripts of recordings were not checked for correctness by human auditors to preserve participant privacy. Words from each participants’ transcripts were stored in randomized order, without any timestamps, to prevent reconstruction of their transcripts, and the audiorecordings were destroyed after transcripts were generated to maintain privacy.

### Analysis

A software tool, Linguistic Inquiry and Word Count (LIWC; version 2015; Text Analysis Portal for Research, University of Alberta) was used to analyze participants’ words along a number of linguistic and psychological dimensions [24]. LIWC is a tool which was developed to categorize words according to both their linguistic function (ie, what part of speech a word is functioning as a noun, adverb, etc) and according to the words’ meanings with respect to psychologically-relevant concepts such as emotions, social concerns, and other constructs. Some of these categories are organized hierarchically, for example, the *affect* category contains the subcategories of positive and negative emotion, and the *negative emotion* category is further broken down into anxiety, sadness, and anger. Examples of these psychological categories, and some of the words within, are given in Table 1.

Participants’ environmental words were analyzed using all possible LIWC categories except summary dimensions, punctuation marks, and informal language. This resulted in 67 total categories that were tested, including the top-level categories of function words (ie, parts of speech), other grammar (ie, more parts of speech), affect, social, cognitive processes, perceptual processes, biological processes, drives, time orientation, relativity, and personal concerns.

Participants who completed all study tasks were included in the analysis if the total number of words detected in their ambient audiorecordings was greater than a minimum of 769 words. This minimum threshold was determined by noting that LIWC was built from a corpus of words, and the least frequently observed word category in the corpus (the sexual words category) had a mean frequency of 0.13% [25]. This implies that, on average, 1 in 769 words in the corpus fell within this category. Assuming that the word data collected from participants are similarly distributed, we would require an expected value of 769 words to detect any words in this category; hence, 769 was the minimum threshold.

**Table 1:** Sample of Linguistic Inquiry and Word Count word categories.

Category	Example words
Personal pronouns	I, them, her
Common verbs	eat, come, carry
Positive emotion	love, nice, sweet
Social processes	mate, talk, they
Death	bury, coffin, kill

The resulting 67 category counts (expressed as the percentage of total words counted which fell within that category) were then tested as correlates of the 4 self-report measures by computing the Pearson correlation coefficient between each category and each measure. Significance of the correlations were tested by computing 2-sided *P* values using the exact distribution of *r*. Due to the exploratory nature of this study, we wished to concisely highlight potentially interesting associations from the large number of correlations measured; therefore, only correlations with an associated *P* value less than .05 are presented. However, due to the large number of comparisons being performed (4 scales × 67 word categories = 268 comparisons), we considered a result statistically significant at a Bonferroni-corrected significance level of α=.0002.

## Results

### Participant Demographics

Of the 112 participants who completed the study, 86 participants yielded sufficient data for analysis. The study sample consisted of 43% females (37/86) and 57% males (49/86), and the average participant age was 30.1 years (SD 8.5). Participant employment status was as follows: 63% (54/86) were employed in full-time work, 16% (14/86) were employed part-time, 12% (10/86) were unemployed and job seeking, 3% (3/86) were not engaged in paying work (eg, retired or homemaker), and 6% (5/86) reported some other employment status. The 86 participants included in analysis and 26 participants excluded from analysis did not differ in mean age, gender distribution, or mean score of any of the 4 self-report measures.

### Self-Report Measures

Table 2 summarizes the self-report measures of the study sample collected at study exit. Intake and exit scores on the LSAS, GAD-7, PHQ-8, and SDS were significantly correlated with *r*=0.90 (*P*<.001), *r*=0.81 (*P*<.001), *r*=0.86 (*P*<.001), and *r*=0.78 (*P*<.001), respectively. We interpreted these strong correlations as indicating the reliability of these measures.

**Table 2:** Results of screening the study sample for depression and anxiety disorders.

Measure	Score, mean (SD)	Diagnostic threshold	Participants over diagnostic threshold (n=86), n (%)
Liebowitz Social Anxiety Scale	53.5 (25.3)	60	32 (37)
Generalized Anxiety Disorder–7	6.5 (4.6)	10	21 (24)
Patient Health Questionnaire–8	8.5 (5.5)	10	30 (35)
Sheehan Disability Scale	10.9 (7.8)	N/A^a^	N/A

^a^N/A: not applicable.

### Environmental Audiorecordings

Within the 86-participant sample, the mean number of audiorecordings captured was 3647 (SD 802), and the mean number of recordings that contained speech was 579 (SD 257). On average, 16% of recorded ambient audio contained intelligible speech. This low percentage is reasonable given that recordings were performed throughout all hours of the day. The average number of detected environmental words per participant was 4379 (SD 2625). While the original transcripts were destroyed after generation, the total number of recordings that contained detected speech was recorded for each participant. The mean number of words was 7.4, which seems reasonable given that the audiorecordings were 15 seconds long. All summary statistics for the total number of recordings captured, number of recordings found to contain speech, total detected words, and average word length of the transcripts are presented in Table 3.

**Table 3:** Summary statistics for word counts of the transcripts of environmental audiorecordings (n=86).

Statistic	Mean (SD)	Minimum	First quartile	Second quartile	Third quartile	Maximum
Total recordings captured	3646 (802)	330	3764	3908	4001	4271
Recordings containing speech	579 (257)	91	390	574	725	1288
Total detected words	4379 (2625)	841	2470	3842	5720	14882
Average number of words in recordings with speech detected	7.4 (2.0)	3.7	6.2	6.8	8.0	15.5

### Correlation Analysis

Table 4 presents the correlations between word counts of the LIWC word categories with each of the 4 self-report measures (LSAS, GAD-7, PHQ-8, and SDS) whose *P* values were less than .05. All 67 categories are presented in Multimedia Appendix 1.

Of the correlations presented in Table 4, only the correlation between the *death* category and PHQ-8 scores was statistically significant (*P*<.001) at a Bonferroni-corrected significance level of α=.0002. This positive correlation shows that higher rates of death-related words detected in the environment are associated with stronger self-reported symptoms of depression.

Interestingly, the rates of words detected in the *positive emotion* and *negative emotion* categories were both measured as having very low associations with all self-report measures, with the absolute value of the Pearson *r* measured under 0.2 in all cases. The rates of words detected in the *negative emotion* category were most strongly correlated with the PHQ-8 (*r*=0.15, *P*=.17). The rates of words detected in the *positive emotion* category were also most strongly correlated with the PHQ-8 (*r*=–0.18, *P*=.09). Correlations and *P* values for all associations, including word rates in the *positive emotion* and *negative emotion* categories, are presented in Multimedia Appendix 2.

**Table 4:** Top correlations between Linguistic Inquiry and Word Count categories and Liebowitz Social Anxiety Scale, Generalized Anxiety Disorder–7, Patient Health Questionnaire–8, and Sheehan Disability Scale scores.

Word category		Percentage of total words, mean (SD)	Correlation, *r*	*P* value
**Liebowitz Social Anxiety Scale**				
	death	0.16 (0.10)	0.32	.002
	home	0.45 (0.14)	–0.31	.003
	see	1.26 (0.28)	0.31	.003
	sexual	0.22 (0.29)	–0.24	.02
**Generalized Anxiety Disorder–7**				
	reward	1.61 (0.30)	–0.29	.007
	death	0.16 (0.10)	0.27	.01
	friend	0.35 (0.15)	0.26	.02
	prep	11.75 (1.10)	0.24	.03
	bio	2.07 (0.59)	–0.23	.04
	relativ	13.57 (1.10)	–0.22	.04
**Patient Health Questionnaire–8**				
	death	0.16 (0.10)	0.41	<.001
	function	55.31 (3.13)	0.24	.02
	home	0.45 (0.14)	–0.24	.03
	reward	1.61 (0.30)	–0.22	.04
**Sheehan Disability Scale**				
	death	0.16 (0.10)	0.28	.009
	friend	0.35 (0.15)	0.24	.03
	negate	2.29 (0.52)	0.23	.03

## Discussion

### Key Findings

A key finding is the correlation between the proportion of detected words within the concept of death and all self-reported measures. This correlation was positive in all cases, meaning individuals who had more death-related words detected in their ambient audio displayed worse self-reported symptoms of social anxiety, generalized anxiety, depression, and mental health-related functional impairment. The association between the use of death-related words and depression is in line with previous studies [26,27] showing that depressed individuals tend to use more death-related words. It is important to note that these prior studies [26,27] analyzed only words that were spoken or written by participants, whereas we included all the words detected in the participants’ environments.

### Other Interesting Findings

In light of the fact that only the correlation between rates of death-related words and the PHQ-8 was statistically significant, it is important to note that the Bonferroni correction is known to be conservative and can cause important relationships to be deemed nonsignificant [28]. That being said, this work has also revealed other interesting potential relationships between different environmental words and mental health.

The first was the positive correlation between vison-related words (the *see* category, including words such as “view,” “saw,” and “seen”) and self-reported symptoms of social anxiety (*r*=0.31, *P*=.003). Higher rates of these words being associated with worse symptoms of social anxiety may be related to a known feature of the disorder. Specifically, individuals with social anxiety disorder fear the scrutiny of others, and socially anxious individuals will attempt to detect this scrutiny by visually attending to the others, especially the faces of others [29]. It may be that individuals verbalize this concern about observing this scrutiny throughout their days.

Another interesting relationship was the negative correlation between the rates of the reward-related words in the environment and self-reported symptoms of generalized anxiety (*r*=–0.29, *P*=.007) and depression (*r*=–0.22, *P*=.04). Lower rates of words in this category, such as “take,” “prize,” and “benefit” were associated with stronger symptoms of generalized anxiety and depression. In the case of depression, this observed association may be linked to the known deficit in reward processing, and therefore, low hedonic tone noted in depressed individuals [30,31]. If the rates of reward-related words can be used as a proxy for reward-seeking, then lower usage rates of reward-related works might be a result of this diminished capacity to focus or search out and respond to rewards. The link between reward and anxiety is less well-understood, but Gray and McNaughton [32] posited that a key feature of anxiety is related to failure or loss of reward. In this sense, anxious individuals may avoid reward-seeking to avoid triggering anxiety related to potential loss of reward. Again, if rates of reward-related works can be used as a proxy for reward seeking, this may shed some light on the observed relationship between reward-related words and symptoms of generalized anxiety.

### Ambient Versus Participant-Only Content Analysis

A key feature of the methodology employed in our study is that the environmental audio recorded for each participant contained speech from any speaker in the environment—the participants themselves but also other humans and recordings (eg, television, radio, music, etc). To the best of our knowledge, no other studies have performed linguistic analysis of audio transcripts containing speech from all ambient sources. This is important to keep in mind when we discuss previous studies that focus only upon speech or writing produced by the participant.

To provide some insight into the impact of other voices in the ambient audio and this study, it is useful to first have an estimate of how much ambient speech is typically produced by the participant and how much comes from other sources. One study [33], which employed a similar audiorecording technology (with wrist-worn smart watches), determined that, of the detected speech in the environment, roughly 18% was produced by the participant, another 18% came from other present people, and 54% from TV and radio. While the presence of other sources of speech in the audio, and therefore in the transcripts, is a confounding factor, it may also contain relevant information. While other individuals will be thought of as polluting the data, the individuals with whom one chooses to associate with may influence one’s own state of mind and mental health, especially with regard to depression [34]. Similarly, the presence of words produced by TV or other media in the environmental audio could be a confound but may also contain useful information. As with the company they keep, participants' choices of media may be reflective of their state of mind and mental health. For instance, one study [35] of film preference and mental health showed an association between preference for film noire movies and depression.

### Comparisons With Other Studies

The most reported association between participant-only word categories and mental health in the literature is the association between the use of first-person personal pronouns and depression. A meta-analysis [11] estimated the correlation to be small (*r*=0.13, 95% CI 0.10-0.16). This correlation was also measured to be quite weak in our study of ambient speech (*r*=0.11, *P*=.30) but with weaker confidence due to a much smaller sample size.

Several studies [36,37] have investigated associations between participant-only linguistic content in social media posts and self-reported measures of anxiety and depression; these same studies have also used LIWC in their analyses and so can be compared with our work. The comparison has the caveat that our work explored speech from other parties in addition to the participant. A linguistic analysis of Facebook posts revealed positive correlations between the *sadness* self-speech word category and self-reported anxiety (*r*=0.34, *P*<.01) [36], whereas our study measured the ambient speech correlation to be much weaker (*r*=0.07, *P*=.51). They also measured the correlation between the *sadness* word category and self-reported symptoms of depression (*r*=0.22, *P*<.01) [36], which corresponds more closely to our results (*r*=0.17, *P*=.13). Another linguistic analysis of Facebook data also found the *sadness* LIWC word category to be a significant predictor of depression diagnosis (standardized regression coefficient β=0.17, *P*<.001) [37].

### Limitations

One technical limitation of this study was the sampling technique used to capture ambient audio. Ambient audiorecordings were produced quite frequently, once every 5 minutes, but for a short duration (only 15 seconds). The short duration of recording helps to preserve smartphone battery life, but it is likely that some conversations or utterances were not captured in full. A more sophisticated sampling technique would record for a variable duration, extending the recording window until silence was detected, so that complete conversations or utterances were captured.

A fundamental limitation is due to the manner in which the environmental audio is used to generate transcripts. Automatic speech recognition software does not perform as well as human transcribers for audio recorded in noisy environments or for audio containing multiple speakers who may be interrupting one another. Furthermore, this software is often being updated and improved; therefore, reproducibility and the ability to do direct comparisons is a key concern for future studies. While this limitation is significant, it is important to also note that the accuracy of Google’s Speech-to-Text API (which was used in this study) has been evaluated in clinical talk-therapy settings and demonstrating 83% sensitivity and 83% positive predictive value in detecting death-related words [38], which implies acceptable validity for the use of this type of data in our analyses.

A final limitation is related to the use of LIWC to perform the linguistic analysis of the transcripts of environmental audio. LIWC is a dictionary-based tool, and as such, categorizes words without looking at contextual information that is key to human language, ignoring sarcasm, metaphor, and analogy.

### Conclusion

This study has explored how the proportions of detected words in ambient speech audio across different grammatical and psychological categories may be associated with self-reported symptoms of social anxiety, generalized anxiety, depression, and general psychiatric impairment. We have highlighted several potential relationships, including associations between death-related words, reward-related word, and words related to vision being potentially associated with self-reported measures of social anxiety, generalized anxiety, depression, and general psychiatric impairment.

## Appendix

Multimedia Appendix 1 Anonymized study data set including scale scores, audiorecording metadata, and LIWC word category percentages for study participants.

Multimedia Appendix 2 All tested correlations (Pearson *r* and *P* values) of LIWC word category usage rates and self-report measures.

## Abbreviations

GAD: Generalized Anxiety Disorder
LIWC: Linguistic Inquiry and Word Count
LSAS: Liebowitz Social Anxiety Scale
PHQ: Patient Health Questionnaire
SDS: Sheehan Disability Scale
